# Design of a High-Power Nanosecond Electromagnetic Pulse Radiation System for Verifying Spaceborne Detectors

**DOI:** 10.3390/s24196406

**Published:** 2024-10-02

**Authors:** Tianchi Zhang, Zongxiang Li, Changjiao Duan, Lihua Wang, Yongli Wei, Kejie Li, Xin Li, Baofeng Cao

**Affiliations:** 1 State Key Laboratory of NBC Protection for Civilian, Beijing 102205, China; 2 College of Information and Communication Engineering, Harbin Engineering University, Harbin 150001, China; 3 School of Electrical Engineering and Automation, Hefei University of Technology, Hefei 230009, China

**Keywords:** high power, nanosecond electromagnetic pulse, radiation system, ionospheric propagation, spaceborne detector verification

## Abstract

The Spaceborne Global Lightning Location Network (SGLLN) serves the purpose of identifying transient lightning events occurring beneath the ionosphere, playing a significant role in detecting and warning of disaster weather events. To ensure the effective functioning of the wideband electromagnetic pulse detector, which is a crucial component of the SGLLN, it must be tested and verified with specific signals. However, the inherent randomness and unpredictability of lightning occurrences pose challenges to this requirement. Consequently, a high-power electromagnetic pulse radiation system with a 20 m aperture reflector is designed. This system is capable of emitting nanosecond electromagnetic pulse signals under pre-set spatial and temporal conditions, providing a controlled environment for assessing the detection capabilities of SGLLN. In the design phase, an exponentially TEM feed antenna has been designed firstly based on the principle of high-gain radiation. The feed antenna adopts a pulser-integrated design to mitigate insulation risks, and it is equipped with an asymmetric protective loading to reduce reflected energy by 85.7%. Moreover, an innovative assessment method for gain loss, based on the principle of Love’s equivalence, is proposed to quantify the impact of feed antenna on the radiation field. During the experimental phase, a specialized E-field sensor is used in the far-field experiment at a distance of 400 m. The measurements indicate that at this distance, the signal has a peak field strength of 2.2 kV/m, a rise time of 1.9 ns, and a pulse half-width of 2.5 ns. Additionally, the beamwidth in the time domain is less than 10°. At an altitude of 500 km, the spaceborne detector records a signal with a peak field strength of approximately 10 mV/m. Particularly, this signal transformed into a nonlinear frequency-modulated signal in the microsecond range across its frequency spectrum, which is consistent with the law of radio wave propagation in the ionosphere. This study offers a stable and robust radiation source for verifying spaceborne detectors and establishes an empirical foundation for investigating the impact of the ionosphere on signal propagation characteristics.

## 1. Introduction

In the field of nanosecond electromagnetic pulse detection, spaceborne detectors are crucial for the identification, timing, localization, and estimation of electromagnetic pulses induced by lightning. These capabilities are essential for disaster prevention and mitigation efforts. In the 1990s, The United States and some countries in Europe proposed a pioneering idea of developing a multi-satellite collaborative network aimed at detecting lightning electromagnetic pulses, which introduced the concept of the SGLLN. This type of research is still in the experimental stage and has made some progress in the single-satellite detection experiment in the low earth [1,2,3,4] and geostationary orbit [5]. However, the randomness and uncertainty of natural lightning events introduce challenges in meeting the calibration and verification needs of SGLLN. While artificially triggered lightning offers precise data over time and coordinate information [6], the intensity and timing of the lightning remain uncontrollable, hindering accurate quantitative calibration. To effectively perform calibration and verification tasks, a ground-based electromagnetic pulse analog transmitter with adjustable power output is necessary. This transmitter comprises a pulse source for signal excitation and a radiation antenna. Given the signal attenuation, spatial dispersion, and absorption by the ionosphere that the analog signal undergoes during propagations, the radiation system must exhibit a high voltage to withstand capabilities in excess of the megavolt level and possess ultra-wideband radiation characteristics within the very high frequency (VHF) band [7,8,9,10]. Meeting these performance criteria poses significant challenges for traditional frequency-domain antennas. Consequently, there is a critical need for research and development focused on designing a radiation antenna based on high-power electromagnetic pulse radiation technology. This endeavor requires innovative engineering solutions to address the demanding requirements of electromagnetic pulse detection systems in the VHF band.

Significant research has been conducted on high-power ultra-wideband (UWB) radiation systems, with a focus on impulse radiating antenna (IRA) and transverse electromagnetic (TEM) horns. The studies have primarily investigated the radiation characteristics, power handling capabilities, and pulse source output performance of these systems. By studying the radiation characteristics of ultra-wideband antennas, some literature has proposed estimation methods for the far-field boundary during experimental measurements [11,12]. A UWB radiation source, named JOLT, has garnered significant attention for its high-performance characteristics, featuring an effective voltage (*rE*) that can reach up to 5.4 MV and a pulse half-width of 100 ps, which has led to its broad application across various fields [13]. To enhance the insulation strength of equipment under high voltage conditions, an innovative approach has been adopted where the antenna is housed within a fiberglass container filled with high-pressure SF6, ensuring the safety and stability of the equipment [14]. Furthermore, researchers have developed a Directive Integrated-Antenna-Source (DIAS), derived from its omnidirectional counterpart, which possesses a 3 dB beamwidth of 234° in the H-plane and 116° in the E-plane, effectively reducing antenna capacitance and spillover loss in the system [15]. Additionally, by integrating a horn antenna with a photoconductive switch, rectangular nanosecond electromagnetic pulses with picosecond rise time can be generated, where the pulse width and rise time are collectively influenced by the length of the system and the excitation pulse waveform [16]. Optimization of the design parameters of the TEM horn antenna, including its size and plate top angle, has been shown to augment the peak field strength and pulse width of the radiated signal in the near-field region [17]. The use of TEM horn antenna arrays also has been proven effective in enhancing the system’s radiation efficiency [18,19]. Recently, researchers have developed an adjustable power impulse radiation system and introduced a method to correct the radiation center using a dielectric lens [20]. These research advancements provide us with valuable insights and indications, but they are predominantly focused on applications such as anti-nuclear damage reinforcement, target identification, and cross-domain interference. Specifically, some of the studies have been constrained to near-field radiation, lacking the high-gain far-field radiation capabilities essential for long-distance propagation. Moreover, they often do not satisfy the nanosecond electromagnetic pulse radiation requirements regarding operational frequency range. These factors render them inadequate for the calibration testing of spaceborne detectors.

In light of these limitations, the present study aims to further explore and optimize the performance of a radiation system to meet the specific demands of spaceborne detector verification. This study is based on a power-adjustable megavolt-level pulse source and a 20 m aperture parabolic reflector, conducting comprehensive research and design from aspects of the feed antenna, insulation structure, protective loading, obstruction assessment, and sensor application. To verify the radiation performance of the system, an experimental phase was implemented, featuring a 400 m far-field test and a 500 km orbit satellite test. Furthermore, our study extends to conducting an in-depth analysis of the characteristics of high-power electromagnetic pulse radiation and its behavior during propagation through the ionosphere.

## 2. Materials and Methods

### 2.1. The Principle of High-Gain Design for TEM Horn Antenna

The TEM horn antenna acts as an impedance-matching device, composed of a pair of triangular conductor plates whose apex angle and the flare angle are adjustable to suit various applications. The far-field radiation intensity along the boresight of the antenna is calculable through analytical methods [21,22]:

(1) $$E(r,t)=\frac{h}{4\pi rcf_{g}}\left\{\frac{dV(t)}{dt}-\frac{c}{2l}\left[V(t)-V(t-\frac{2l}{c})\right]\right\}$$

In (1), *V*(t) represents the excitation signal, *c* is the speed of light, *r* is the axial distance from the horn, *l* is the axial projection length of the plate, *h* is the horn height, *w* is the horn width, and *f*_g_ is the structural impedance factor, which is defined as the ratio of the horn’s characteristic impedance *Z*_c_ to the free-space wave impedance *Z*_0_ (approximately 377 Ω, *f*_g_ = *Z*_c_/*Z*_0_). The characteristic impedance *Z*_c_ is related to the aspect ratio *h*/*w* of the horn structure and can be derived using the transmission line theory [21].

(2) $$Z_{c}=\left\{\begin{array}{l} 120\ln\left(\frac{4h}{w}+\frac{w}{2h}\right)\;\;\;\;\;\;\;\;\;\;\;\;\;,\;\frac{h}{w}>1\;\\ 240\pi /\left(\frac{2w}{h}+1393+0.667\ln(\frac{2w}{h}+1.444)\right),\;\frac{h}{w}\leq 1\; \end{array}\right.$$

Reference [23] has proposed the time-domain gain *G*_r_ to characterize the radiation performance of a transient antenna, defined as:

(3) $$G_{r}=\frac{2\pi \sqrt{f_{g}}\left|rE_{\mathrm{rad}}\left(\theta ,\varphi ,t\right)\right|}{\left|dV_{\mathrm{inc}}\left(t\right)/dt\right|}$$

This definition expands the traditional notion of antenna gain into the time domain, facilitating the assessment of the antenna’s response to transient signals. In (3), *E*_rad_ (*θ*, *ϕ*, *t*) represents the radiated electric field in the far-field region, dependent on spherical coordinates *θ* and *ϕ*, and time *t*; *V*_inc_ is the incident voltage. By combining Equations (1) and (3), the time-domain gain can be simplified to:

(4) $$G_{r}=h/2\sqrt{f_{g}}$$

**Figure 1:**
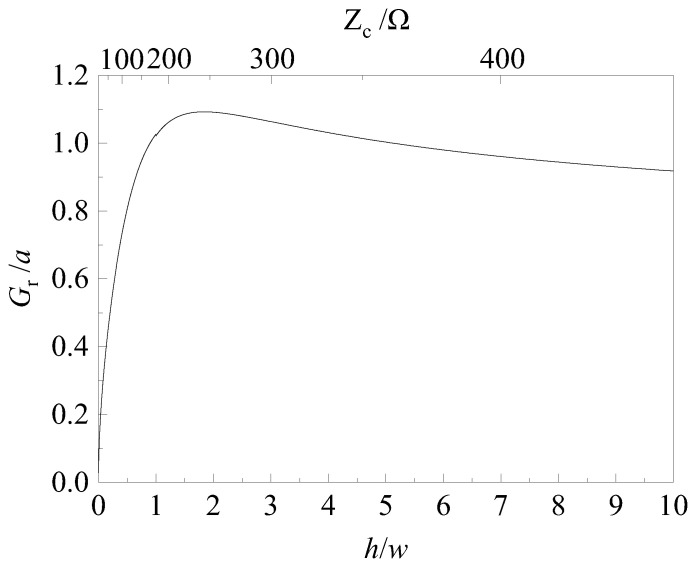
The time-domain gain of TEM horn antenna.

In order to achieve high-gain radiation performance within a limited space, this research confines the aperture of the TEM horn to a circumference defined by a radius *a*. By employing Equations (2) and (4), the variations in time-domain gain *G*_r_ with respect to the aspect ratio *h*/*w* and characteristic impedance *Z*_c_ are depicted in Figure 1. It is observed that the time-domain gain increases initially as both the aspect ratio and characteristic impedance of the horn aperture are augmented. Subsequently, the gain decreases after reaching a peak, with a pronounced maximum occurring at a characteristic impedance of approximately 280 Ω, corresponding to an aspect ratio of about 1.84.

### 2.2. The Optimization of the TEM Antenna Size Parameters

**Figure 2:**
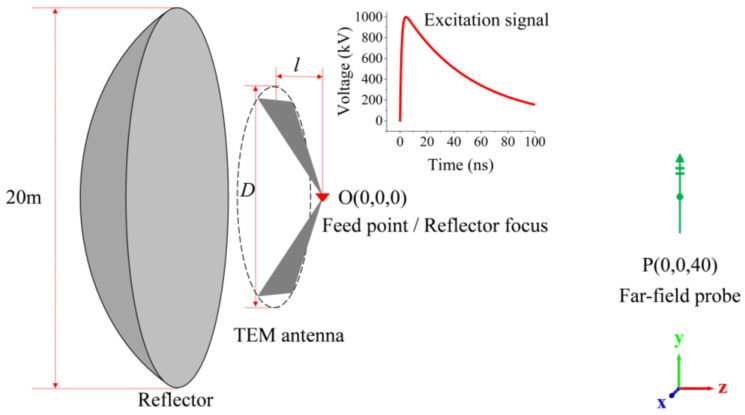
Schematic diagram of the simulation layout.

The simulation method in this paper is based on the Time-Domain Finite Integration Technique (TD-FIT), and the modeling and computation are carried out using the CST Studio Suite^®^ 2021. According to the analysis in Section 2.1, the primary structural parameters of the TEM horn antenna include the projection length of the plate, as well as the height and width of the horn’s aperture. In engineering applications, the TEM horn antenna needs to work in conjunction with a 20 m aperture reflector. Thus, while keeping the projection length of the antenna on the Z-axis (*l* = 2 m) and the aspect ratio (*h*/*w* =1.84) constant, we vary the circumcircle diameter *D* (m) of the aperture to study the waveform variation in the far-field region. During this simulation process, the excitation signal utilizes a double-exponential pulse waveform with an amplitude of 1 MV, a rise time of 2 ns, and a pulse half-width of 40 ns. Figure 2 is the schematic diagram showing the layout of the TEM horn antenna, the 20 m aperture reflector, and the far-field probe. In the cartesian coordinate system, the feed point of the TEM horn antenna is located at the focal point O (0,0,0) of the reflector, and driven by a discrete port. The far-field probe is positioned at point P (0,0,40), which is 40 m away from the feed point.

**Figure 3:**
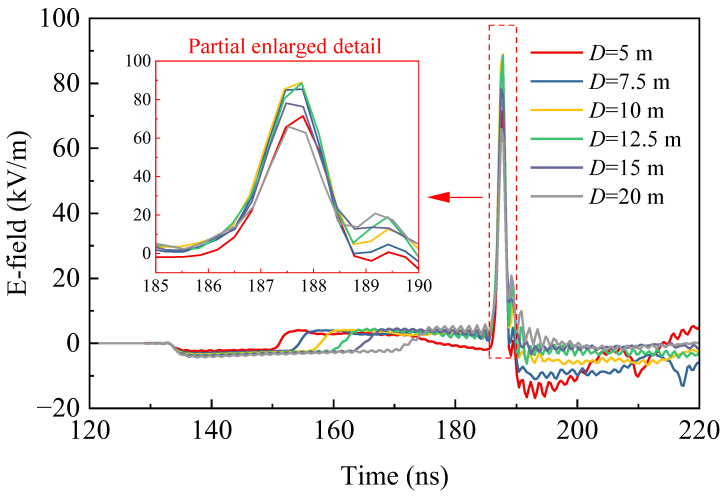
Simulation results of the far-field probe.

Figure 3 presents the simulation results of the far-field radiation waveforms for different parameters *D* (5, 7.5, 10, 12.5, 15, 20 m). The results indicate that as the value of *D* increases, the peak field strength of the radiation field first increases and then decreases, while the pulse half-width and rise time are not significantly changed. The improvement is attributed to the appropriately increased size of the TEM horn antenna, which enhances radiation efficiency, consequently boosting the peak field strength of the radiated field. However, an excessively large antenna size can lead to issues: on one hand, it may increase the flare angle of the horn antenna, which could reduce the directivity of the radiation and cause energy leakage; on the other hand, it can create a more significant blocking effect in the radiation field, resulting in a gain loss. Therefore, the size of the TEM horn antenna’s aperture—both in terms of height and width—must be carefully selected to avoid dimensions that are either too small or excessively large, as these could have a negative impact on the desired peak field strength of the radiation field.

Based on the simulation results, the size of the TEM horn antenna has been determined as follows: the height *H* of the horn aperture is 9 m, and the width *W* is 5 m. The height *H*_0_ of the horn feed part is 0.8 m, and the width *W*_0_ is 0.43 m. To enhance the performance of the radiation antenna, the sectional width *w*(z) and height *h*(z) of the horn antenna along the z-axis are designed with an exponential gradient, as expressed by the following equations:

(5) $$\left\{\begin{array}{c} w(z)=W_{0}e^{\frac{z}{L}\ln(W/W_{0})}\\ h(z)=H_{0}e^{\frac{z}{L}\ln(H/H_{0})} \end{array}\right.$$

**Figure 4:**
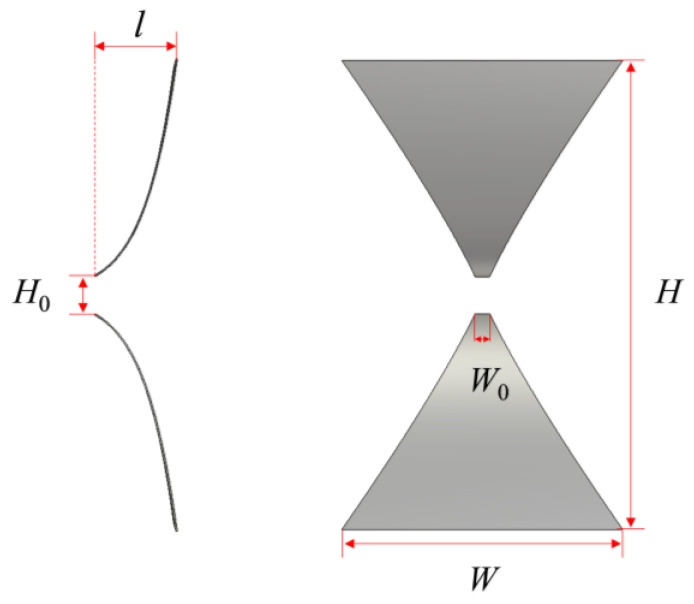
Diagram of the TEM horn antenna structure.

The structural configuration of the TEM horn antenna is depicted in Figure 4, illustrating its optimized dimensions and the cross-sectional geometry defined by an exponential gradient.

### 2.3. The Electromagnetic Pulse Source and Pulser-Integrated Antenna

**Figure 5:**
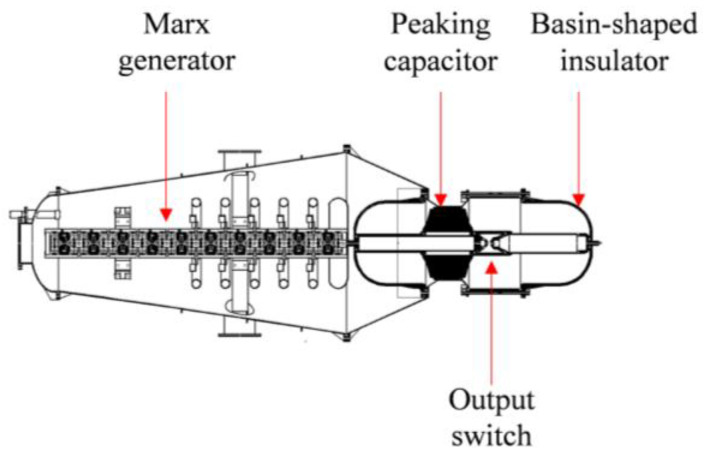
Diagram of the pulse source structure.

**Figure 6:**
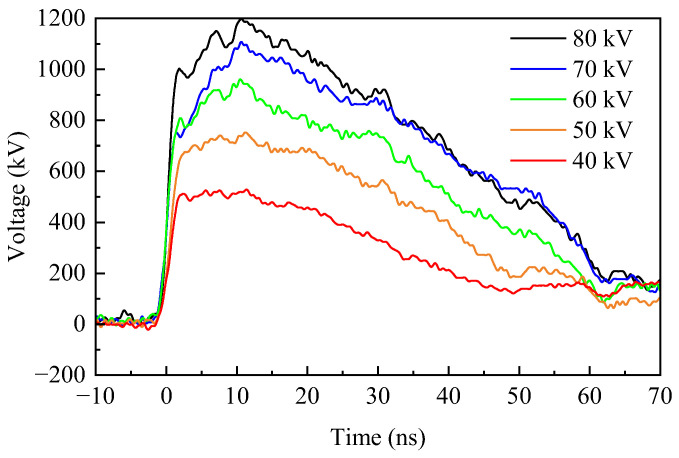
The output waveforms of the pulse source at different charging voltages.

The electromagnetic pulse source serves as an energy provider for the radiation system, which predominantly consists of a Marx generator, a peaking capacitor, and an output switch, among other components. Figure 5 presents a schematic depiction of this configuration. The Marx generator is arranged in a linear configuration, where the output voltage is amplified through 20 cascading stages to produce a pulsed output after charging the energy storage capacitors in parallel. The pulse source is capable of modulating the parameters of the output voltage waveform by varying the charging voltage of the energy storage capacitors and adjusting the pressure of the insulating gas. Additionally, it possesses variable frequency output capability, adjustable from 1 to 10 Hz. Figure 6 illustrates the output voltage waveforms corresponding to different charging voltages of the energy storage capacitors. The waveforms exhibit a clear double-exponential pulse trend, with essentially the same rise time and pulse half-with across different curves, averaging around 1.9 ± 0.3 ns and 39 ± 0.7 ns, respectively. The peak voltage of the waveform is positively correlated with the charging voltage and can reach levels in the megavolt range.

**Figure 7:**
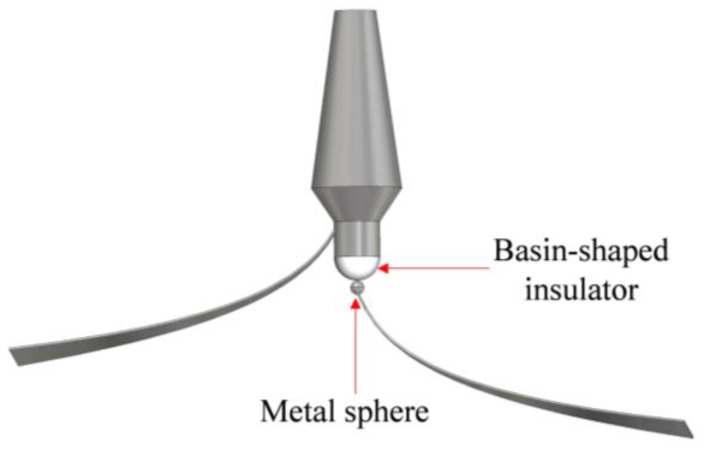
Diagram of the pulser-integrated antenna.

In the design of the connection structure, considering the high output voltage of the pulse source, we depend on the basin-shaped insulator for making connections. As shown in Figure 7, a plate of the TEM horn is connected to the central position of the insulator (the positive pole output), with a metal sphere added to enhance the uniformity of the electric field distribution. The other plate is connected to the negative pole and maintains a safe distance from the outer edge of the insulator. Ultimately, the feed antenna has become a configuration of pulser-integrated antenna. This design eliminates the energy loss associated with coaxial transmission structures and reduces the high-voltage breakdown risk posed by external balun structures.

**Figure 8:**
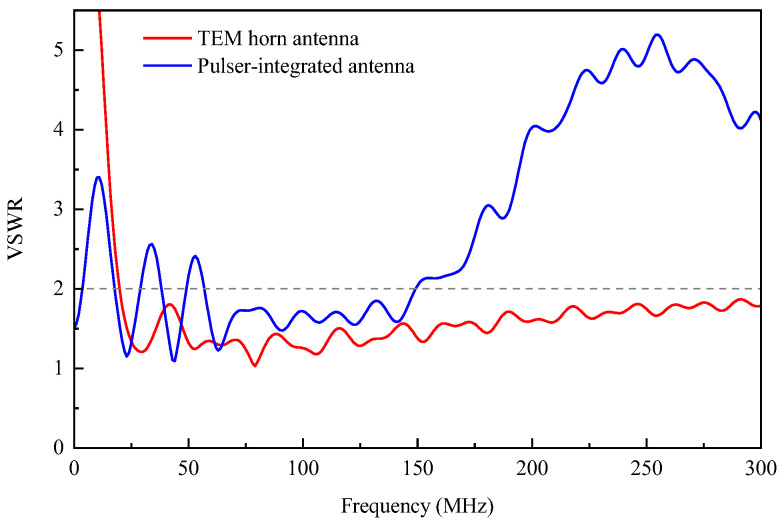
Simulation results for the VSWR.

To study the impact of the feed antenna structure variations on the operating frequency band, simulations were conducted for both the TEM horn antenna and pulser-integrated antenna to calculate the voltage standing wave ratio (VSWR). The simulation results presented in Figure 8 indicate that, under the same VSWR conditions, the operating bandwidth of the pulser-integrated antenna is somewhat reduced compared to that of the TEM horn antenna. However, it exhibits good impedance matching at frequencies below 200 MHz, with a VSWR of less than 3 in the range of 14 to 190 MHz, and even better matching with a VSWR of less than 2 in the range of 57 to 149 MHz, thus meeting the requirements for VHF ultra-wideband radiation performance.

The power capacity of a high-power system is primarily limited by phenomena such as arc discharge, dielectric voltage breakdown, or corona discharge. The breakdown voltage of gas under nanosecond electromagnetic pulses is significantly higher compared to that under steady-state voltages, indicating a characteristic overvoltage breakdown. An empirical relationship, as summarized by TH Martin [24], is expressed in the following equation:

(6) $$\rho \tau =97800{\left(E_{b}/\rho \right)}^{-3.44}$$

In this equation, *ρ* (g/cm³) denotes the gas density, *τ* (s) signifies the duration of the action, and *E*_b_ (kV/cm) represents the average breakdown field strength. At standard atmospheric pressure, the air density is approximately 1.25 × 10^−3^ g/cm³. By considering the action time as 90% of the peak average pulse width of the output signal from the pulse source (approximately 12.8 ns), we can calculate the critical electric field strength *E*_b_ = 48.5 kV/cm by substituting these values into the equation. Therefore, the maximum withstand voltage of the system, *V*_max_ = *E*_b_ × *H*_0_, is estimated to be 3.88 MV.

### 2.4. The Asymmetric Design of Protective Loading Mechanism

When operating under high-power conditions, the TEM horn antenna, being in an open circuit state at its termination, often generates a substantial reflected voltage. This reflected voltage will affect the operating frequency of the antenna and have a negative impact on the pulse source. The harmful reflected voltage can generally be mitigated by loading resistance at the termination of the horn, which is typically performed either at the front or back using symmetrical loading methods. The loading schemes studied in Reference [17] include a method that connects 4 resistors in parallel at the back of the horn, effectively reducing voltage reflection. However, this paper utilizes a working mode where the main radiation direction of the system is opposite to that of the horn. Based on the principle of magnetic electric dipoles, the front loading of the horn is more conducive to enhancing the forward gain of the reflector. To determine the optimal loading positions, based on TD-FIT, we have added a surface current monitor (type of time domain) in the simulation model to observe the current distribution on various metal surfaces.

**Figure 9:**
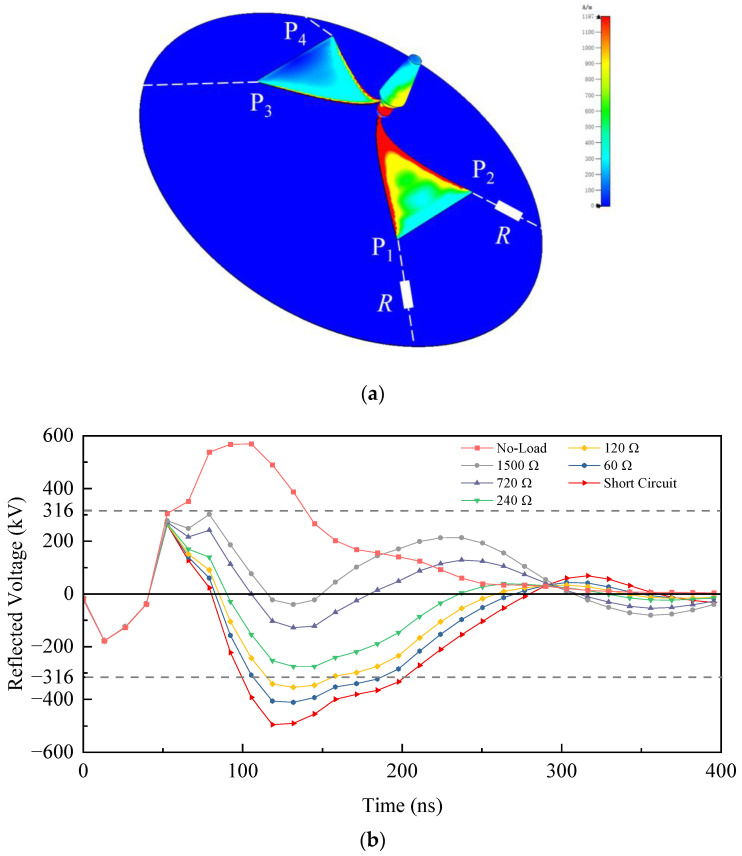
Simulation results of the radiation system: (**a**) Surface current; (**b**) Reflected voltage.

The results shown in Figure 9a indicate that the current distribution on the horn is mainly concentrated at the edges of the plates, with a higher current density observed on the positive plate compared to the negative plate. This suggests considering an asymmetrical loading design at the front of the horn. Based on the above analysis, it is planned to connect 2 resistors *R* in parallel at the top points P_1_ and P_2_ of the positive plate of the horn and connect them to the reflector. Meanwhile, the top points P_3_ and P_4_ of the negative plate will be short-circuited to the reflector through wires in order to create a surge current loop, thereby reducing the reflected voltage. The proposed loading scheme effectively preserves the positive gain generated by the current loop while ensuring unobstructed radiation.

In order to quantitatively describe the reflection voltage of the system, we conduct a simulation analysis on the reflected voltage at the excitation port location. The simulation result in Figure 9b demonstrates that the peak reflected voltage of the no-load system can reach 576 kV, with an amplitude exceeding 316 kV—equivalent to approximately 10% of the excitation signal’s peak power—around 85 ns into the signal. This level of reflected voltage poses a significant threat to system security, particularly concerning the stable operation of the pulse source. The simulations have been conducted to optimize parameters across a range of resistance *R* in the loading schemes. The results indicate a high degree of consistency in the reflected voltage waveforms among different resistance *R* within the initial 50 ns of signal injection. However, approximately 50 ns later, noticeable waveform divergence occurs among different *R* values, and the amplitudes of the waveforms undergo oscillatory decay. As detailed in Table 1, a decrease in the resistance *R* led to a reduction in the maximum positive peak amplitude and a corresponding increase in the maximum negative peak amplitude. When the resistance *R* ranges from 240 Ω to 1500 Ω, the peak voltages of both polarities remain below the 316 kV. According to the principle of minimizing reflected energy, when the resistance *R* is set to 720 Ω, the total reflected energy over a 400 ns interval is calculated to be 18.1 J, representing a reduction of 78.9% compared to 85.7 J in the no-load system. This optimization significantly enhances the operational safety and stability of the device.

### 2.5. The Assessment of the Obstruction Effects on the Radiation Field

In the study of designing the feed antenna for a reflector, it is crucial to consider the obstruction effects caused by the physical structure and their influence on radiation gain. Reference [25] discusses the obstruction effects based on the forward projected area of the feed antenna. However, due to the complex structure of the feed antenna, it establishes a nonlinear relationship with the scattering field effect, which significantly limits the applicability of this method. In order to address the problem, the paper proposes an innovative assessment method for quantifying obstruction gain loss of the feed antenna, based on the principle of Love’s equivalence in electromagnetism [26]. This method provides a more nuanced understanding of the antenna’s performance in relation to obstruction effects.

Initially, construct a suitable square enclosure that fully encloses the feed antenna structure, designated as the S-surface. During the simulation of the antenna’s excitation, record the distribution of electric field **E**(**r**′) and magnetic field **H**(**r**′) on the S-surface. These field data can be converted into surface current (**J**_s_ = **n**′ × **H**(**r**′)) and surface magnetic current (**K**_s_ = **E**(**r**′) × **n**′) through differential operations, and where **n**′ is the unit vector in the direction normal to the S-surface. According to the principle of Love’s equivalence, when using these equivalent field sources for excitation, the radiated field maintains the same result beyond the region of the S-surface. The magnetic field **H***^e^*(**r**) and the electric field **E***^e^*(**r**) generated by a surface current source, and the electric field **E***^m^*(**r**) and the magnetic field **H***^m^*(**r**) generated by a surface magnetic current source are as follows:

(7) $$\left\{\begin{array}{c} H^{e}\left(r\right)=\frac{1}{4\pi }\nabla \times \oint_{s}\frac{n^{\prime }\times H\left(r^{\prime }\right)}{\left|r-r^{\prime }\right|}e^{-jk\left|r-r^{\prime }\right|}dS^{\prime }\\ E^{e}\left(r\right)=\frac{1}{j\omega \varepsilon }\nabla \times H^{e}\left(r\right)\\ E^{m}\left(r\right)=-\frac{1}{4\pi }\nabla \times \oint_{s}\frac{E\left(r^{\prime }\right)\times n^{\prime }}{\left|r-r^{\prime }\right|}e^{-jk\left|r-r^{\prime }\right|}dS^{\prime }\\ H^{m}\left(r\right)=-\frac{1}{j\omega \mu }\nabla \times E^{m}\left(r\right) \end{array}\right.$$

where *k* is the wave number (*k* = 2π/λ), *j* is the imaginary unit. The total field generated by the equivalent field source is the superposition of the radiation fields produced by the current source and the magnetic current source:

(8) $$\left\{\begin{array}{c} E\left(r\right)=E^{e}\left(r\right)+E^{m}\left(r\right)\\ H\left(r\right)=H^{e}\left(r\right)+H^{m}\left(r\right) \end{array}\right.$$

**Figure 10:**
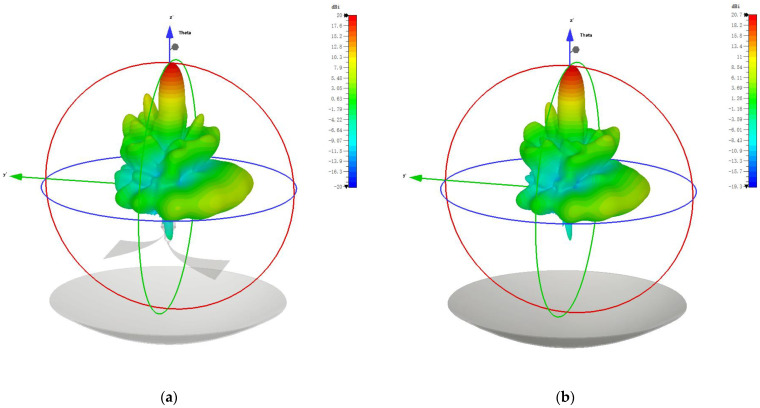
The simulation results at 100 MHz: (**a**) Radiation pattern excited by the feed antenna (obstruction present); (**b**) Radiation pattern excited by the equivalent field source (no obstruction).

In the case of the radiation field reflected by a reflector, the equivalent field source only supplies the excitation signal without having a physical presence, thus precluding any obstruction effects. This allows for an assessment of the feed antenna’s obstruction gain loss by comparing its far-field performance under two different excitation methods. Based on the TD-FIT, the simulation process needs to be carried out in two parts. Since the equivalent field source information can only be simulated for a specific frequency point, the first step is to select a series of frequency points within the frequency band of interest (we chose 25 MHz, 50 MHz, 100 MHz, 150 MHz, 200 MHz, 250 MHz, and 300 MHz; a total of seven frequency points). In this step, we solve for the equivalent field source through numerical computation while saving the field source information. In the second step, we establish the reflector model and import the seven field sources’ information obtained from the previous simulation. Then, we activate another round of numerical simulation to obtain far-field radiation gain simulation results of the model. Taking a simulation frequency of 100 MHz as an example, Figure 10 illustrates the simulation results for the far-field radiation patterns. The simulation results indicate that there are some differences in the far-field patterns between the two excitation methods, attributable to obstruction effects.

Table 2 presents simulation outcomes across a broad frequency range, showing an upward trend in gains with increasing frequency, regardless of the presence of obstruction effects. The average gain excited by the feed antenna across different frequencies is 20.7 dBi, and for the equivalent field source, it is slightly higher at 21.6 dBi. Additionally, the obstruction effects of the feed antenna result in different gain losses across frequencies, with the highest loss observed at 25 MHz (1.47 dB), the lowest loss at 50 MHz (0.32 dB), and an overall average loss of approximately 0.9 dB. Therefore, it can be concluded that the obstruction effects of the feed antenna on the radiation field have little impact on the performance of the radiation system.

### 2.6. The Sensor for High-Power Electromagnetic Pulse Measurement

**Figure 11:**
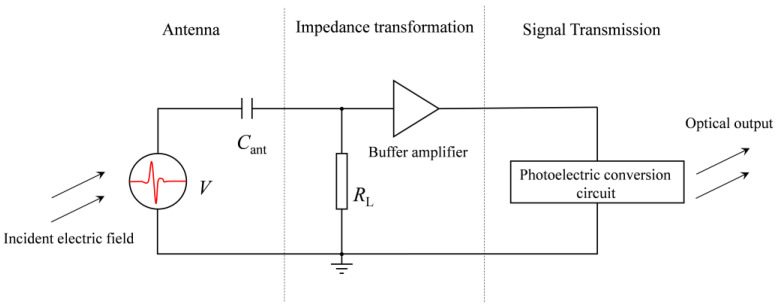
The circuit schematic of the E-field sensor.

**Figure 12:**
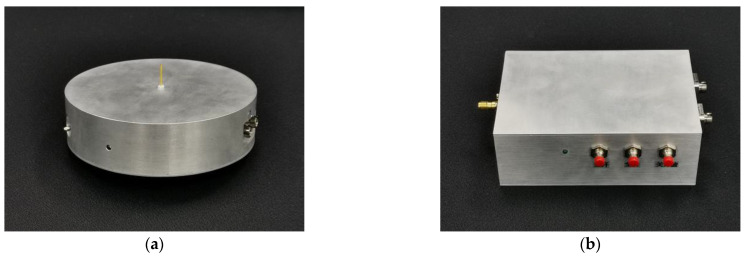
The photos of the measuring equipment: (**a**) E-field sensor; (**b**) Photoelectric converter.

In the development and utilization of radiation systems, it is essential to effectively and accurately measure the radiation field. Considering the high peak field strength, rapid rise time, and extended radiation range of the electromagnetic pulse radiation system [17], we utilize an E-field sensor to measure the time-domain waveform of the pulsed electric field and employ fiber optic transmission to enhance the signal’s resistance to interference. The sensor, which consists of an antenna, a signal conditioning circuit, and an optical transmitter, constitutes the core part of the entire measurement system. The receiving antenna utilizes an electrically small antenna with a monopole cylindrical shape. After the antenna captures the electric field signal, it undergoes impedance transformation and signal conversion before being connected to a fiber-optic link in the back end. In order to improve measurement performance, high-impedance JFET operational amplifiers (with an impedance of 500 GΩ) and surface-mount resistors and capacitors are used in the E-field sensor. The shielding enclosure is designed in a disc shape, providing excellent portability and environmental adaptability. The relevant circuit schematic and physical photos are shown in Figure 11 and Figure 12.

**Figure 13:**
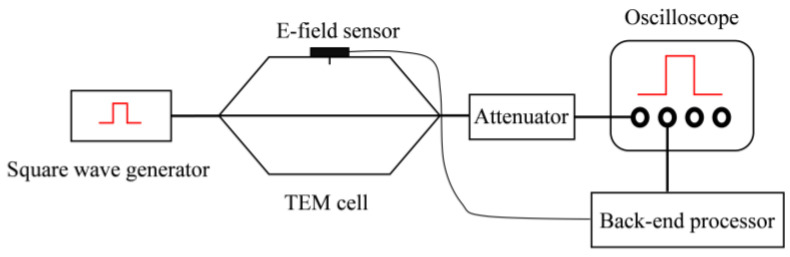
The diagram of sensor calibration experiment configuration.

We used a TEM cell to calibrate the E-field sensor. To avoid affecting the uniform electric field distribution inside the cell after placing the sensor, we inverted the E-field sensor above the TEM cell with its front shell aligned with the inner surface of the cell. The schematic diagram is shown in Figure 13.

**Figure 14:**
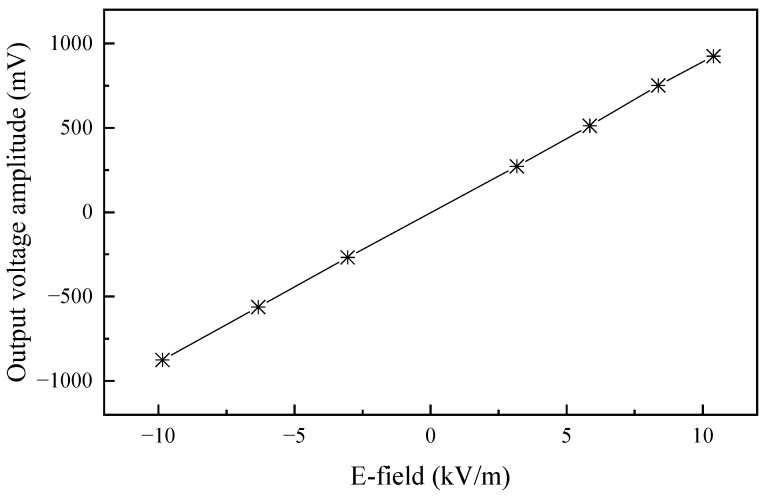
Calibrated sensitivity curve of the E-field Sensor.

**Figure 15:**
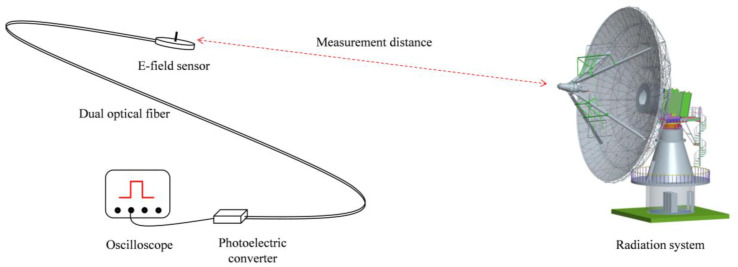
The diagram of the measurement system.

The sensitivity curve obtained from the calibration experiment is shown in Figure 14, which reveals that the sensor demonstrates excellent linear consistency in terms of sensitivity. However, due to the thermal drift effect in the electro-optical conversion process of the semiconductor laser in the sensor, its light emission efficiency generally decreases with the increase in temperature. Additionally, changes such as bending or replacement of the optical fiber, as well as variations in the tightness of the optical fiber plug, can all result in fluctuations in optical power. These factors make it difficult for the sensitivity coefficient established after calibration to remain constant, thereby affecting measurement accuracy. To address this, we have added a standard square wave signal for calibration in the sensor and adopted a dual optical path transmission mode. One path is responsible for measuring the electric field signal, while the other is responsible for detecting the square wave signal. This design allows the sensor to perform functions such as real-time sensitivity calibration and circuit continuity checking during the testing process. See Figure 15.

During usage and testing, the calibration square wave amplitude U_1_ is first read. The relative change in standard square wave amplitude (U_0_/U_1_) is proportional to the change in optical power, and the corrected current system sensitivity coefficient can be obtained as S_1_ = S_0_ × (U_0_/U_1_). This method enables calibration of the entire sensor chain, effectively improving measurement efficiency and accuracy.

## 3. Results

**Figure 16:**
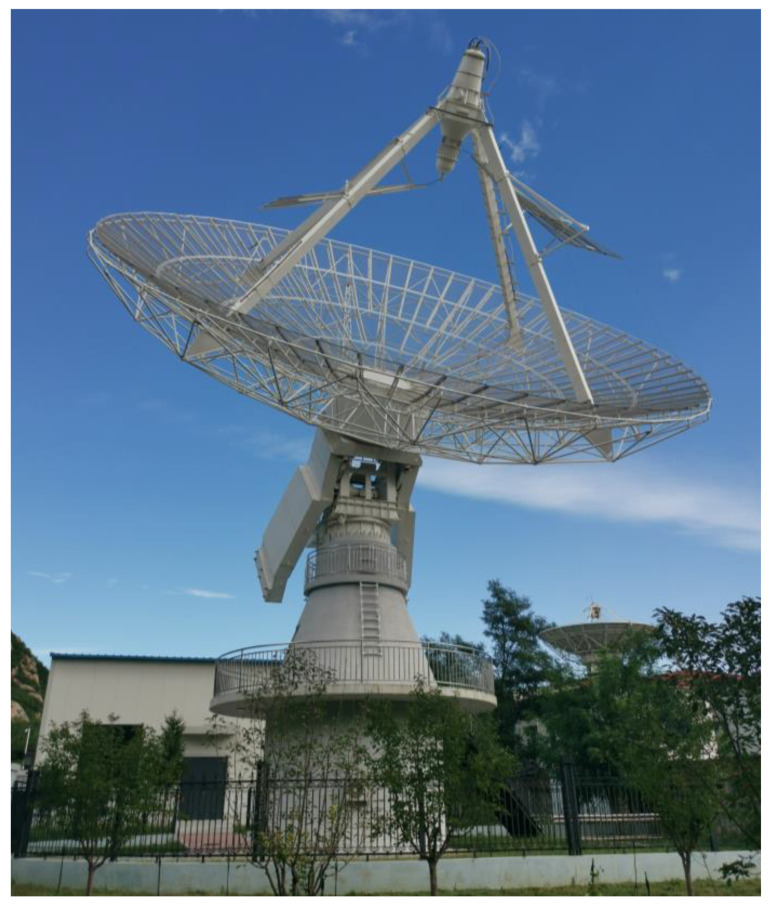
The photograph of the electromagnetic pulse radiation system.

The electromagnetic pulse radiation system, as shown in Figure 16, is constructed with a fully rotatable pedestal structure that enables high-precision servo control in polarization, azimuth, and elevation directions. In the content of this chapter, we have completed the ground-based far-field testing experiment and the space-based on-orbit testing experiment, respectively.

### 3.1. Testing in the Far-Field Region at a Distance of 400 Meters

When conducting performance testing for a UWB radiation device, it is necessary to study their far-field conditions based on phase changes, and the minimum distance for far-field measurements must adhere to the formula: *r* ≥ 2 *D*_a_^2^ /λ_min_ [11]. Given the aperture of the reflector (*D*_a_ = 20 m) and the simulated operating frequency range (*f*_H_ ≤ 150 MHz, λ ≥ 2 m), it is deduced that the minimum measurement distance required to evaluate far-field performance should exceed 400 m.

**Figure 17:**
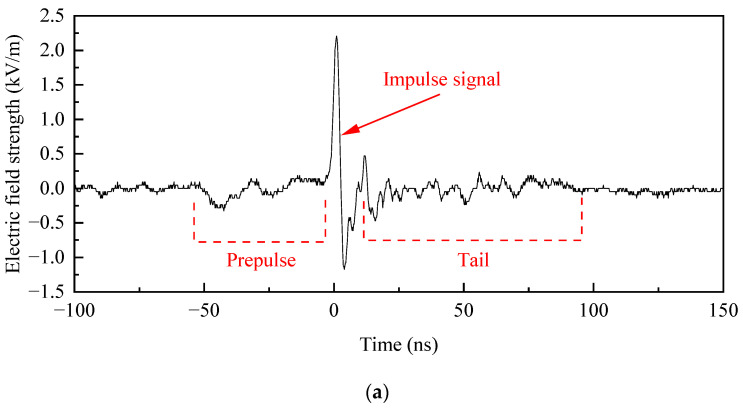

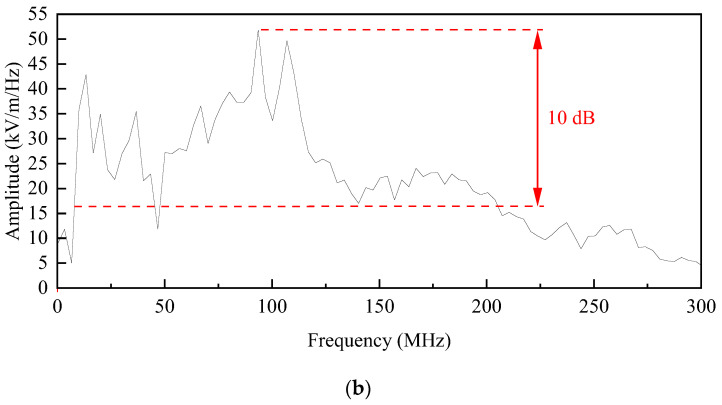
Radiating signal in the far-field region at a distance of 400 m: (**a**) Time-domain waveform; (**b**) Frequency spectrum.

In order to ensure the continuous operational stability of the device and minimize electromagnetic pollution, a single-shot emission mode of the pulse source is used during the ground-based testing experiment, with a charging voltage of 50 kV and a measurement distance of 400 m. The specified sensor described in Section 2.6 is employed for conducting the experiment. As shown in Figure 17a, the main pulse waveform exhibits clear bipolar features, with a rise time of 1.9 ns, a pulse half-width of 2.5 ns, a peak field strength of 2.2 kV/m, a peak-to-peak field strength of 3.4 kV/m, and an effective voltage of 880 kV. Furthermore, an initial pre-pulse of about 53 ns duration is detected prior to the impulse signal, which is attributed to the backward radiation effect of the feed antenna. After performing a Fast Fourier Transform (FFT) on the time-domain waveform, we obtained the frequency spectrum of the signal, as shown in Figure 17b. This spectrum exhibits a central frequency of 94 MHz, with a 10 dB bandwidth spanning from 8 to 205 MHz. The relative bandwidth is calculated at 185%, and the in-band energy constitutes 91%. The spectral distribution of the signal aligns with the standard characteristics of VHF ultra-wideband signals. Due to the capacitor charging voltage being set at only 50 kV during testing (the maximum value can reach 100 kV), there is significant room for increasing the effective voltage of the electromagnetic pulse signal radiated by the system at full power.

**Figure 18:**
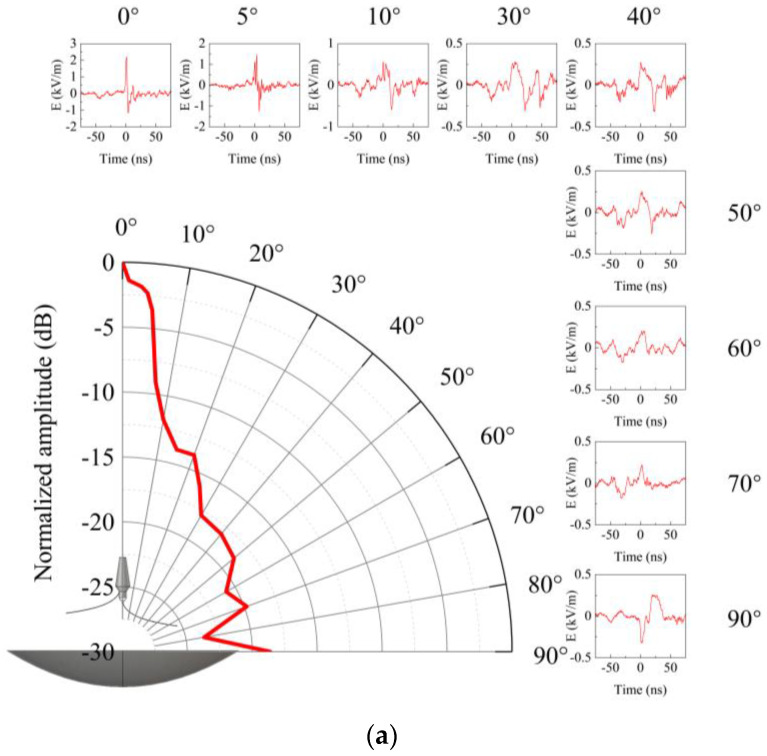

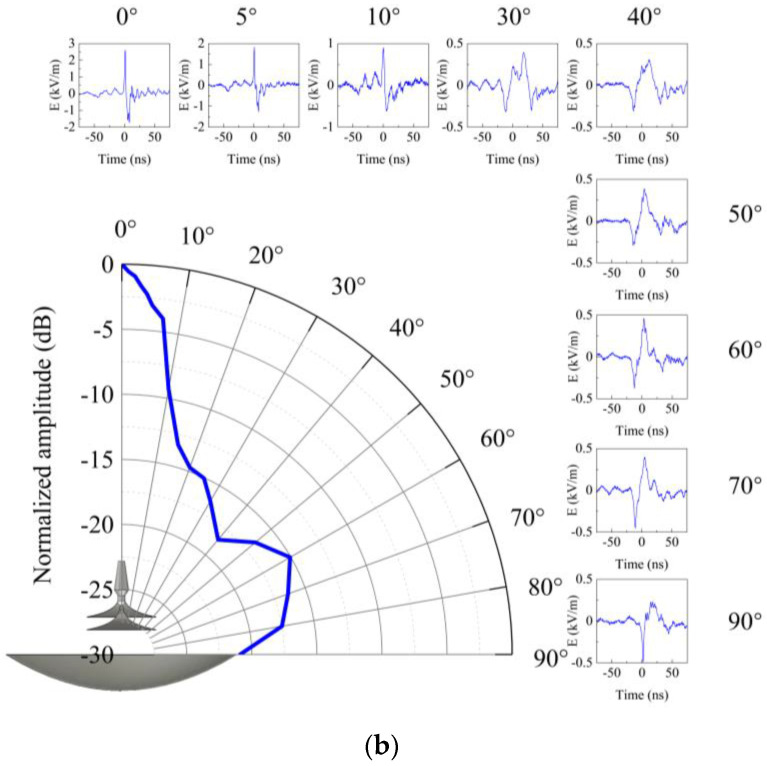
Time-domain radiation patterns and partially selected signal waveforms: (**a**) E-plane; (**b**) H-plane.

To conduct a thorough study on the performance of the radiation system across various emission angles, precise control of boresight deviation angles within the servo constraints, and the waveforms in two orthogonal polarization planes were recorded. The time-domain radiation patterns, based on peak field strength, along with selected partial-signal waveforms, are presented in Figure 18.

According to Figure 18, as the deviation angle from the boresight increases from 0° to 40°, the peak field strength of the impulse decreases while the pulse half-width expands; within the angle range of 40° to 90°, the waveform of signal exhibits complexity due to the reflection and obstruction effects. However, there is a discernible increase in the pre-pulse amplitude with increasing angle. The time-domain radiation pattern reveals pronounced main lobe features in both the E- and H-plane, with the 3 dB beamwidths of 9.1° and 9.9°, respectively, and the side lobe levels are consistently below −15 dB.

### 3.2. Testing in the On-Orbit Satellite at an Altitude of 500 km

In the free space, the group velocity of electromagnetic waves is equal to the speed of light (*c*), and it is constant for all frequencies. However, on the propagation path to the satellite, the free electrons and ions in the ionosphere interact with electromagnetic waves, causing different frequency components of the signal to propagate at different group velocities, thereby causing signal dispersion. Some papers are dedicated to establishing the model of electromagnetic pulse propagation through the ionosphere [27,28,29]. These studies prove that the dispersion effect distorts the UWB electromagnetic pulse and changes its time-frequency characteristics, resulting in significant energy attenuation and broadening of pulse duration.

On 13 November 2023, at 07:20 Beijing time, we conducted the on-orbit testing experiment at the Beijing test site (approximately located at 40° N, 116° E), with the satellite involved in the experiment having an orbital altitude of 500 km [4]. At this moment, the elevation angle of the radiation device is 87.4°, and the line-of-sight distance to the satellite is approximately equal to the orbital height of the satellite. The ionosphere parameters can be viewed in Appendix A, according to the ionosphere parameters from the International Reference Ionosphere (IRI) database [30]. In order to enhance the signal detection efficiency, we increased the charging voltage of the pulse source to 60 kV and adjusted the emission frequency to 2 Hz during the experiment.

**Figure 19:**
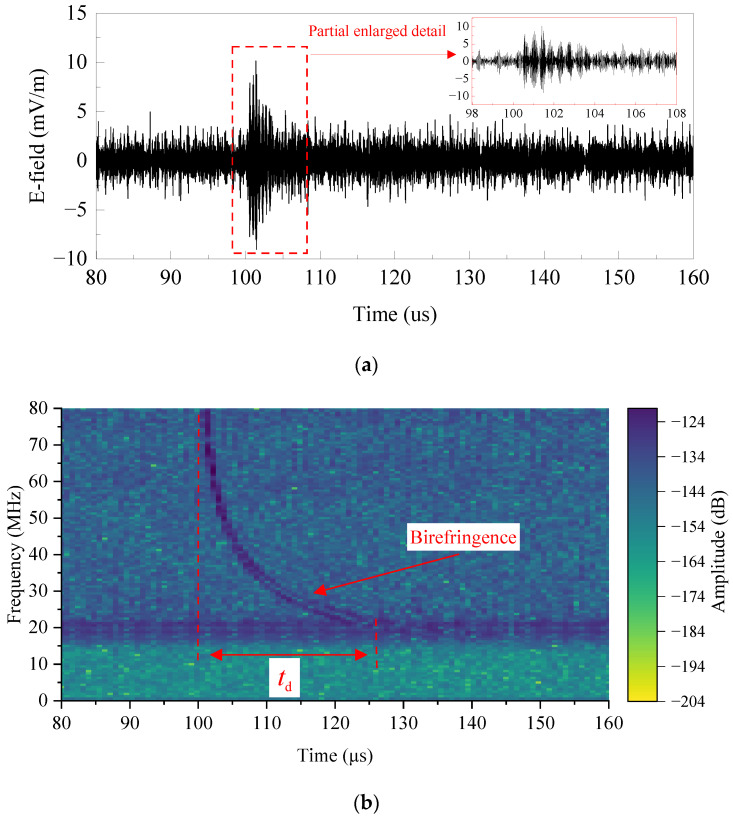
Radiating signal at 500 km satellite orbit: (**a**) Time-domain waveforms; (**b**) Time–frequency analysis.

The spaceborne detection results are shown in Figure 19. Specifically, Figure 19a illustrates an oscillating signal with a duration spanning tens of microseconds and a peak field intensity of approximately 10 mV/m, which significantly differs from the characteristics of the nanosecond electromagnetic pulse transmitted by the radiation system. The Short-Time Fourier Transform (STFT) is employed for further signal analysis. The time–frequency curve in Figure 19b clearly shows typical dispersion and birefringence phenomena. The UWB signal has transformed into a nonlinear frequency-modulated signal with a duration of approximately 25 microseconds, which is primarily caused by the dispersive effect of the ionospheric plasma, and the lower the frequency, the greater the impact of dispersion. Additionally, the signal gradually divides into two components with closely spaced frequencies below approximately 30 MHz. This is due to the presence of the geomagnetic field, which gives rise to a different influence on the ordinary (“O”) wave and extraordinary (“X”) wave components within linearly polarized waves, resulting in distinct propagation speeds in the ionosphere (the “O” wave propagates faster than the “X” wave). Consequently, a gradual division phenomenon occurs between the two wave components, and as the frequency decreases, this separation becomes more pronounced. During practical application, the signal should undergo additional scientific processing to meet the calibration requirements of spaceborne detector experiments.

## 4. Discussion

To highlight the novelty and advantages of our proposed design scheme, in Table 3, we summarize a comparison of the measured results from our work and other high-power electromagnetic pulse radiation equipment. In the cited references [19,20], the peak voltage of the pulse sources does not exceed 100 kV, which inherently limits their capacity to achieve higher output power for radiation. The radiation fields described in references [13,15] exhibit significant *rE* values, yet their pulse widths, at only a few hundred picoseconds, do not extend into the nanosecond range. This characteristic indicates a higher frequency spectrum, rendering the signals unsuitable for applications in spaceborne detector testing experiments. The equipment detailed in reference [17] exhibits a radiation field with nanosecond-level pulse width and rise time in measurements. However, the operational region of this antenna is limited to the near-field of radiation. Therefore, even with an increase in the voltage amplitude of the pulse source, the equipment is still unable to effectively radiate into the far-field region.

The radiation system proposed in this paper has a rise time of 1.9 ns, a pulse half-width of 2.5 ns, and an effective voltage of 880 kV in the far-field region, with a signal 10 dB bandwidth ranging from 8 to 205 MHz. This is attributed to the high-gain optimization of the feed antenna, in conjunction with a sizable 20 m diameter reflector antenna, endowing the equipment with far-field radiation capability for lower frequency signals. Furthermore, the integrated design has enhanced feeding efficiency and withstand voltage strength. The implementation of an asymmetric loading technique reduced the reflected voltage, thereby equipping the equipment with superior power capacity and safety stability.

## 5. Conclusions

This article presents the design of a radiation system capable of emitting high-power nanosecond electromagnetic pulses, detailing the optimization processes aimed at enhancing the time-domain gain, feed efficiency, and reducing reflected voltage. An innovative assessment method is proposed to evaluate the obstruction loss of the feed antenna in the radiation field. This article also introduces a specialized sensor for measuring strong electric fields, and successfully applies it to the measurement of the testing experiment. The results indicate that the radiation system achieves an average gain of approximately 20.7 dBi within the VHF band, with gain losses kept below 1.5 dB across all frequency points. Experimental tests have been carried out in a far-field region at 400 m and on a satellite orbit at an altitude of 500 km. The far-field radiation of the equipment reaches an effective voltage of 880 kV, with a pulse half-width of 2.5 ns, and a time-domain beamwidth of less than 10°. Despite propagation losses and the effects of ionospheric absorption and dispersion over hundreds of kilometers, the pulse signal remains effectively detectable by a spaceborne detector, and its time–frequency characteristics conform to the physical laws governing electromagnetic pulse propagation through the ionosphere.

The high-power nanosecond electromagnetic pulse radiation system serves as an experimental platform for calibrating and verifying spaceborne detectors. It allows for adjusting the radiation power by varying the output voltage of the pulse source, enabling precise quantitative calibration of the detectors. The development and application of this study can effectively enhance the detection accuracy of SGLLN, particularly in determining the location and intensity of the lightning, which plays a significant role in thunderstorm warning and disaster risk reduction efforts.

## Figures and Tables

**Table 1:** Simulation results for reflection voltage of different schemes.

Different Schemes	Maximum Positive Peak Value (kV/m)	Maximum Negative Peak Value after 50 ns (kV/m)	Total Reflected Energy within 400 ns (J)
No-Load	575.7	/	85.7
R = 1500 Ω	305.2	−81.3	29.6
R = 720 Ω	290.0	−138.5	18.1
R = 240 Ω	280.1	−288.7	28.3
R = 120 Ω	277.0	−361.2	45.1
R = 60 Ω	275.3	−416.4	60.1
Short Circuit	273.6	−502.9	83.1

**Table 2:** The results for the far-field gain of the radiation system.

Frequency	Obstruction Present (dBi)	No Obstruction (dBi)	Gain Loss (dB)
25 MHz	5.91	7.38	1.47
50 MHz	14.88	15.20	0.32
100 MHz	20.02	20.66	0.64
150 MHz	21.08	22.31	1.23
200 MHz	25.06	25.97	0.91
250 MHz	27.58	28.75	1.17
300 MHz	30.28	31.08	0.80

**Table 3:** Comparison of the performance among different radiation equipment.

Ref.	Type of Antenna	Approximate Size (m)	Radiated Impulse Signal			Region of Interest
			*rE* (kV)	Rise Time (ns)	Pulse Half-Width (ns)	
[13]	Half-Impulse Radiating Antenna	3 × 1.5 × 1.2	5300	0.08	0.1	Far-field region
[15]	Integrated Feed Antenna with Reflector	1.5 × 1.5 × 0.6	1580	0.15	0.25	Far-field region
[17]	TEM Horn Antenna	3 × 3 × 3	26.9	2.5	20.9	Near-field region
[19]	Four-Element Array TEM Horn Antenna	0.4 × 0.4 × 0.6	340	0.06	0.08	Far-field region
[20]	Impulse Radiating Antenna	1.2 × 1.2 × 0.42	44	0.33	0.43	Far-field region
This article	Integrated Feed Antenna with Reflector	20 × 20 × 25	880	1.9	2.5	Far-field region

**Figure A1:**
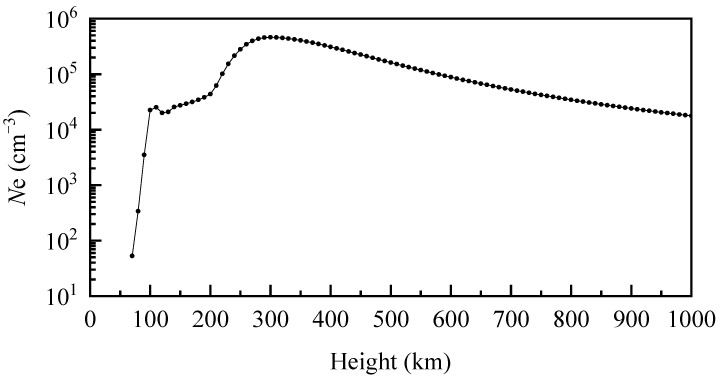
Altitude profiles of electron density.

## Data Availability

Data are contained within the article.

## Appendix A

When conducting space-based on-orbit testing experiments, ionospheric parameter data are crucial for our research and analysis of the propagation characteristics of electromagnetic pulses. Figure A1 describes the electron density of the ionosphere at altitudes below 1000 km for that location and time.

Based on Figure A1, it can be observed that at an altitude of 300 km, the electron density is highest, with *N*_e_ = 4.61 × 10^5^ cm^−3^. At this point in time, the critical frequency of the ionosphere can be calculated using the following equation:

(A1) $$2\pi f_{p}=\sqrt{\frac{N_{e}e^{2}}{\varepsilon_{0}m_{e}}}$$

resulting in a critical frequency *f*_p_ = 6.1 MHz. By performing a path integral of electron density, we obtain the total electron content (TEC) for this path as 9.28 × 10^10^ cm^−2^.
